# Access Improvement in Healthcare: Be Quick, but Don't Hurry?

**DOI:** 10.1002/lrh2.70052

**Published:** 2025-12-05

**Authors:** Allen M. Chen

**Affiliations:** ^1^ Department of Radiation Oncology, University of California, Irvine Chao Family Comprehensive Cancer Center Orange California USA

**Keywords:** access, health policy, health services, leadership, strategy

## Abstract

**Background:**

Access improvement is a fundamental component of value‐based healthcare as it inherently promotes quality by eliminating chokepoints, redundancies, and inefficiencies that could hinder the provisioning of timely care. Yet as healthcare organizations struggle with cost containment, the question of how to most effectively enhance access remains largely unsolved. Given the economic, regulatory, and social forces across the healthcare marketplace, the critical importance of access in optimizing efficiency is increasingly being recognized. The purpose of this review is to thus present a practical framework that offers healthcare organizations an actionable, thematic‐based foundation for approaching access improvement.

**Methods and Materials:**

An interpretive synthesis of health access as it relates to the timely, satisfactory, and sustainable receipt of services was presented while addressing operational constraints, inclusivity and underlying determinants of health. The criticality of this concept, spanning the entire healthcare continuum and encompassing all aspects of care delivery, from making an initial appointment to completing treatment and being followed thereafter, was evaluated. Empirical lessons were highlighted for discussion.

**Results:**

Given the sense of urgency that exists around this issue, the potential pitfalls of “hurrying” to initiate access improvement are frequently overlooked by health systems. Considerations related to workforce shortages, resource limitations, logistical coordination, workflow processes, space capacity, provider availability, and organizational culture and hierarchy, among others, need to be methodically addressed so as not to introduce new inefficiencies into a healthcare environment that is already viewed by many as overly bureaucratic. Based on the core themes that emerged, a conceptual framework for access improvement centered on strategy, alignment, execution, adaptation, and reflection was developed.

**Conclusion:**

The design, planning, and operationalization of access improvement initiatives in healthcare require meticulous organizational preparation and a deliberate leadership approach incorporating principles of change management. Success will be dependent on achieving the appropriate balance between speed, purpose, and precision.

## 1 Introduction

The rhetorical phrase—“be quick, but don't hurry—” is attributed to iconic college basketball coach John Wooden and was worded to motivate his players to optimize speed and agility on the court while simultaneously maintaining discipline, self‐control, and focus on team effort [1]. Although outwardly paradoxical, this leadership‐inspired adage takes on special consideration in modern healthcare given the importance of patient access. The present reality is that wait times for health services are longer than ever, and excessive delays in securing appointments represent a major conundrum for patients across all segments of society [2]. As Salisbury et al. recently conveyed, a “waiting crisis” has emerged in many developed countries where lags in accessing health services can number weeks if not months [3]. This is despite the increasing evidence showing that timing matters in healthcare [4].

Studies from across conditions have shown that delays in care can be detrimental, leading to worsening and/or progression of disease without timely diagnosis and treatment [5]. Furthermore, difficulties with appointment scheduling can result in mounting frustration for many patients, contributing to anxiety, depression, and apathy—leading to an overall sense of helplessness or even depersonalization. It is thus evident why patients consistently rate access as one of the most important factors affecting their satisfaction with the healthcare system [6].

In a previous study, a 12‐step framework for addressing access improvement was constructed based on a systemic review of the literature [7]. In the current work, a practical narrative is presented delving into key leadership lessons that can be extracted from the prior exercise—with the goal of outlining empirical strategies on how operational progress can be achieved.

Given the need to ensure that patients are seen as expeditiously as possible, health systems are increasingly focused on access improvement. However, making strides in access is not as straightforward as it might appear on the surface. Relatedly, it is important to recognize that access by itself is not synonymous with quality. For instance, simply expediting appointments without thoughtful consideration of the downstream implications with respect to appropriateness has the potential to lead to compromises in delivery even leading to wasteful, hastily administered, and/or substandard care, all of which are of low value. In this sense, “hurrying” to institute access improvement, despite the best of intentions, should be avoided, particularly in view of the complex and multifaceted nature of this concept. Indeed, the potential perils of “hurrying” to address access improvement are rampant and generally involve concerns related to staffing shortages, infrastructure limitations, logistical coordination, space capacity, and provider availability, among others.

The rush to improve access thus poses the real possibility of introducing new inefficiencies and burdens to health care systems already perceived as overly bureaucratic rather than rectifying the ones that any initiative so idealistically aims to address. Moreover, it is increasingly obvious that social determinants heavily influence the provisioning of health services. Neglecting crucial factors like affordability, geographic accessibility, cultural competency, and education including health literacy, all of which can significantly impede individuals from actually utilizing services even when they are technically available, has the potential to undermine efforts to improve access.

So, how should the aphorism “be quick, but don't hurry” really be interpreted? While Coach Wooden intuitively differentiated between quickness and hurrying, the distinction, in actuality, is much more difficult to grasp. In practical terms, what Coach implied is that the more one hurries, the more likely that focus is lost, leading to mishaps and errors—and inefficiency. However, at the same time, without quickness, any sense of purpose can be diluted, resulting in the surrendering of any competitive edge needed to achieve eminence. Although these teachings were initially meant for basketball (and also, life), they are equally applicable to the “game” of healthcare. Similar to basketball, healthcare is dependent on an organization's ability to react to external circumstances with fluidity while maintaining focused control and effort—particularly when it pertains to clinical quality improvement. With respect to patient access, the need to maintain balance—assuring that details are not overlooked while understanding the larger big picture—is critical to make meaningful and sustainable gains. The purpose of this report is to thus provide a practical, interpretive framework based on the “be quick, but don't hurry” mantra for approaching access improvement in healthcare.

### 1.1 Preparation

At the most basic level, the ability to satisfy the needs of an expanding patient population depends critically on ensuring that the appropriate infrastructure is in place. From a simplistic viewpoint, this is a matter of supply‐and‐demand, as a viable foundation for growth is a prerequisite for enhancing access. This is because resource limitations related to personnel, physical space, and/or provider availability can stifle even the most ambitious access improvement programs by creating patient backlogs and longer waits. Thus, the importance of expanding provider capacity—through provider recruitment, training of more qualified physicians, and/or reliance on non‐physician extenders such as nurse practitioners, physician assistants, and patient navigators—is unquestioned. As such, the development of appropriate and innovative staffing models, considering such factors as competitive compensation, external benchmarking, and community engagement, is crucial. In the face of lingering shortages, one potential solution is to develop training programs that could serve as a grooming ground and pipeline of talent; yet another is to provide incentives, both financial and non‐financial, to prospective providers. Although recruitment and workforce development can be notoriously laborious, they represent worthwhile investments for any health system aiming to expand access and frequently require creative and unconventional planning. The utility of telemedicine platforms, in certain situations, has also been demonstrated to alleviate provider shortages. Digital health can also serve as a means to promote patient engagement through enhanced communication, education, and awareness of health‐related issues [8]. Acknowledging that digital health is a broad, multidisciplinary term encompassing platforms, systems, and services that use information and communication technologies to improve health and healthcare outcomes, how to best apply such tools in practice remains uncertain. However, generally accepted priority areas for health systems include those focused on cybersecurity, electronic health record modernization, revenue cycle management, and the leveraging of advanced analytics and machine learning to improve efficiency and productivity. Other high‐priority areas center on augmenting patient access and experience through promoting virtual health, automating back‐office functions, and developing remote patient monitoring and hospital‐at‐home programs to address labor shortages and enhance care delivery. Furthermore, as society grows in diversity, the importance of creating a workforce that reflects its varying races and ethnicities is also recognized. Finally, the need to proactively address provider burnout is also essential to minimize turnover and maintain staffing levels. Initiatives focused on preserving staff morale and building workplace wellness can be valuable in this regard. As the workforce grows, the optimization of physical space takes on greater importance. Because mismatches between utilization and demand can create inefficiency, the need to identify and reallocate underutilized clinic rooms is imperative. Given that access improvement centers on extending services to meet a seemingly growing demand, thoughtful preparation to ensure that an organization is not stretched too thin is critical to planting the seeds of success in advance.

### 1.2 Cohesion

A prerequisite for attaining the delicate balance between being quick and hurrying is ensuring that alignment exists across all stakeholders towards a common objective. First, the promotion of a shared vision starts with organizational commitment—and clear and transparent messaging regarding the rationale for access improvement is critical to garner effective buy‐in from all employees. The goal herein is to not only create a sense of unified purpose but to engender outward pride around access improvement. Given that access improvement will frequently require a dramatic shift in culture, numerous aspects of workflow will need to be dissected and refined, with the goal of enhancing efficiency so that slack can be built to accommodate capacity. In this regard, a collaborative, “all hands” approach is imperative. Targeted, consistent, and data‐driven communication strategies thus provide the context for personnel to understand the why, what, and so what of any strategy being considered. To build support, open forums should be devised specifically to raise concerns and scrutinize proposals, especially with how to attain balance between accommodating enhanced access while maintaining normal operational activity. As administrative tasks related to insurance, authorization, and billing are related to access, the engagement of financial staff can be useful to gain insight into the intricacies of how patients historically enter the health system and to identify any barriers. Since ancillary staff including nursing, front‐desk support, medical assistants, schedulers and greeters are typically most familiar with clinic workflow—ideas to enhance patient throughput which can free up access should be solicited. The utility of dedicated coordinators to identify low‐value, inappropriate, and/or unnecessary visits which could be eliminated thereby creating room to expand access should also be highlighted. Furthermore, investing in navigators, scribes, and resource specialists can serve to streamline workflow, as these support staff can tackle much of the mundane, administrative aspects of clinical care, thus allowing physicians to focus on direct medical management. Lastly, the importance of physician leadership, particularly with respect to providing insight into its practical integration into clinical workflow, cannot be understated. Opportunities to leverage artificial intelligence (AI)‐based solutions for documentation, diagnostic evaluation, and clinical decision‐making should be explored. For example, the development of effective transitions of care templates using AI can greatly facilitate coordination of care, particularly when triaging and/or handoffs are necessary [9]. Overall, for any access improvement initiative to be successful requires finding that cohesive balance so that all team members are working as one with common expectations, purpose, and accountability.

### 1.3 Execution

Access improvement in healthcare is not as straightforward as allowing every patient who wants an appointment to obtain one at their whim. Instead, the focus on efficiency is paramount. Given this distinction between expediency and efficiency, while acknowledging the overlap, the goals of access improvement might vary between institutions that face differing challenges. For instance, the immediate needs of patients from rural areas are commonly dissimilar from those located in urban or suburban regions. Relatedly, differences in what constitutes an acceptable wait time might differ between specialists and/or primary care providers. Regardless of these distinctions, the general goal of access improvement is to minimize delays in obtaining health services for patients and to empower individuals to take charge of their own healthcare. In this regard, initiatives termed “advanced access scheduling” have been proposed to offer more flexible and patient‐centric options for appointment acquisition [10]. Although this concept has taken on varying definitions, ranging from open scheduling utilizing patient‐specific online self‐service portals to the provisioning of same‐day or next‐day appointments, it has generally been heralded as a step in the right direction with access improvement [11, 12, 13]. The prospect of extending clinic availability to provide appointments that might be more convenient for the working population outside of normal 9–5 h has also been considered. Furthermore, urgent care centers and retail clinics are emerging as access options allowing patients to connect to care outside of a provider's office hours. The establishment of mobile clinics in “healthcare desserts,” or geographical areas traditionally devoid of healthcare services has also been shown to be effective. Digital communication tools are another means of reaching patients who might not otherwise have access. The introduction of “digital front doors,” which are user‐friendly interfaces designed to facilitate the sharing of information and to promote access has been shown to improve the patient experience [14, 15]. The use of telemedicine, electronic patient portals, mobile health apps, AI‐based virtual navigators, and online health information resources, has soared in recent years and has increasingly been integrated by healthcare organizations into patient‐care platforms. These technologies offer novel opportunities to promote patient engagement and to communicate with patients from a wider range of demographics, regardless of geographic location, employment status, socioeconomic hierarchy, or educational background—and to bridge access gaps in care on an instantaneous basis. Furthermore, the deployment of health‐related wearables and mobile technology has contributed to more efficient ways of dynamically monitoring patients for a variety of conditions outside of the hospital. Given the increasing recognition of the role that social determinants play in health, patients should also be screened to identify at‐risk groups with respect to housing, transportation, and social support. Lastly, it is critical that efforts to improve societal engagement and to address inequities focus both on immediate gaps such as income and social status, as well as on factors more “upstream” such as early childhood education and wellness including physical activity, nutrition, and violence prevention. In this sense, access improvement should have the potential to be scalable on a broader, community‐based setting, in order to truly reach the masses. In this sense, being quick means approaching the problem of access from all angles and setting a commitment to meaningful change to have the most sustained impact over time.

### 1.4 Monitoring

Both in basketball and in healthcare, scorecards and dashboards incorporating a variety of quantitative and qualitative metrics are increasingly used to evaluate every aspect of the environment, particularly with respect to performance. Assessing patient access should be no different. However, identifying which metrics should be monitored in this regard can sometimes be uncertain. For instance, for rural hospitals, commonly assessed parameters include those related to childhood vaccination, primary care catchment, and/or cancer screening, whereas, in more populated regions, the focus might be on appointment waits for designated specialists and/or the time elapsed prior to the reply of patient messages through digital portals. Regardless of the specific setting, an abundance of key performance indicators has been proposed for clinical practice including access‐related benchmarks such as third‐next‐available appointment, time from referral to appointment, office wait time, patient call handle time, no‐show percentage, appointment cancellation rate, and results from patient experience surveys. The assimilation of analytics and development of real‐time dashboards related to scheduling capacity, staffing optimization, space utilization, cost‐effectiveness, and supply chain considerations have the ability to truly take access metrics to the next level, thereby helping to facilitate value‐based care. Moreover, the analysis of potential associations between access statistics and commonly used quality metrics, including those related to survival, is also critical to achieve a better understanding of the impact on timely care and health outcomes. For instance, one goal of access improvement is to decrease the rates of re‐hospitalization and unnecessary emergency room utilization by providing more readily available options for patients to seek care in an ambulatory setting. The utility of access improvement to bridge traditionally observed disparities in healthcare should also be explored, and metrics focused on health equity are urgently needed. The harnessing of AI‐based tools also has the potential to transform data analytics with respect to access improvement. For instance, the data acquired during routine care can be inputted into machine learning models to create algorithms to assist with patient flow and to identify barriers to access optimization. Moreover, studies have shown that AI can predict patients who are at risk for no‐shows and/or use digital portals and can assist with scheduling processes by smartly considering such factors as space, provider time, staffing, and demand based on historic patterns [16]. Such algorithms can result in not only enhanced access and throughput, but also drive improved financial performance by eliminating wasteful expenses. Ultimately, the goal of any tracking system is to ensure that data‐driven decisions are made based on the continual analysis of real‐time information extracted from both patient and organizational measures including those related to utilization, effectiveness, efficiency, and satisfaction—so that actionable steps can be taken for process improvement. In more specialized situations, the comparison of the acquired data to national benchmarks and standards can be useful when these exist.

### 1.5 Pivot

As with any team sport such as basketball, healthcare is critically dependent on making fine‐tuned adjustments to changes—expected and unexpected—in a reflective and purposeful manner. In this sense, the importance of change management cannot be understated. For instance, limitations or constraints may need to be imposed if access improvement leads to growth that is overly taxing on the workforce. Conversely, thoughtful introspection will be required if the proposed initiative does not immediately have its desired effect of improving access. Regardless of the scenario, the failure to strategically respond to problems can damage morale, undermine culture, and/or sqaunder managerial investment. In this sense, devising opportunities for stakeholders to participate in decision‐making or to provide feedback can spur organizational engagement that can facilitate course correction when needed. The inclusion of patient advocacy groups can also help provide perspective and potential solutions for snafus that develop. Since uneven usage of any access improvement initiative can lead to more pronounced inequities in care, adjustments will often be required to address this predicament. Similarly, barriers related to insurance and authorization can often be magnified as access improvement initiatives are developed locally at the level of the health system. Consequently, creating a sense of alignment with payors through dialogue, particularly within the context of value‐based care delivery models, can help establish critical support that can mollify hurdles. The importance of community‐based engagement can also identify solutions that help the organization move past chokepoints that impede access. For instance, the use of transportation vouchers and/or ride‐share programs can be useful for patients who have difficulty accessing providers even when appointments are available due to logistical circumstances [17]. Furthermore, enhanced community awareness can identify gaps that could be addressed through targeted initiatives focused on improving health education, peer support, and digital literacy. Given the increased recognition of the concept of “financial toxicity” that burdens many patients, the relationship between access and healthcare‐related expenses can also be addressed as access improvement initiatives mature [18]. For instance, financial counselors have been shown to significantly improve the patient experience by reducing financial distress and anxiety, which in turn can positively impact health outcomes and satisfaction [19]. They provide crucial support by helping patients navigate costs, understand their insurance, and find financial assistance, which allows patients to focus more on their treatment and recovery. This role is vital for patient well‐being, especially given that financial worries are a major source of stress for many individuals undergoing medical care. Furthermore, efforts to promote price transparency have the potential to break down many practical barriers which stymie access [20]. By empowering consumers (i.e., patients) to make informed decisions, conduct comparisons across the marketplace, and avoid financial surprises, price transparency promotes access. Although pivots should never be taken lightly, adjustments in strategy are invariably required, even if minor, in the development of quality improvement initiatives. Given that access improvement will often be met with initial skepticism and/or viewed as a fancy marketing gimmick more in line with padding the financial pockets of the organization than with an earnest effort to enhance the patient experience, responsiveness and flexibility will be necessary. Ultimately, adjusting course when needed will allow for the sustainability of any initiative and to keep up with the pace of change as it evolves.

## 2 Conclusion

As outlined in the present work, the practical implementation of any access improvement effort is not simply about “doing more with less” but involves achieving a critical balance between speed and thoughtfulness. Given that many systems are already operating in ‐financially constrained environments, the need to focus on identifying system‐based inefficiencies and to conduct a comprehensive operational assessment—addressing resource utilization, workforce productivity, labor expenses, supply costs, staffing levels, payor strategy, revenue cycle management, and other key areas—should be prioritized. Recognizing that variability exists in workflow across practices based on geography, employment model, patient population, funding, and scope, among other considerations, a “one‐size‐fits‐all” solution will generally not be sufficient. The successful implementation of any access improvement program will thus require meticulousness in the functional analysis of every step of the patient journey through the care continuum, while simultaneously considering external factors related to social determinants. In this regard, access improvement for healthcare organizations, analogous to the game of basketball, requires systematic planning, careful coordination, and steady leadership. Most notably, both involve a heterogeneous amalgamation of moving pieces and are thus vitally dependent on the cohesiveness of the team, as well as purposeful coaching.

**FIGURE 1:**
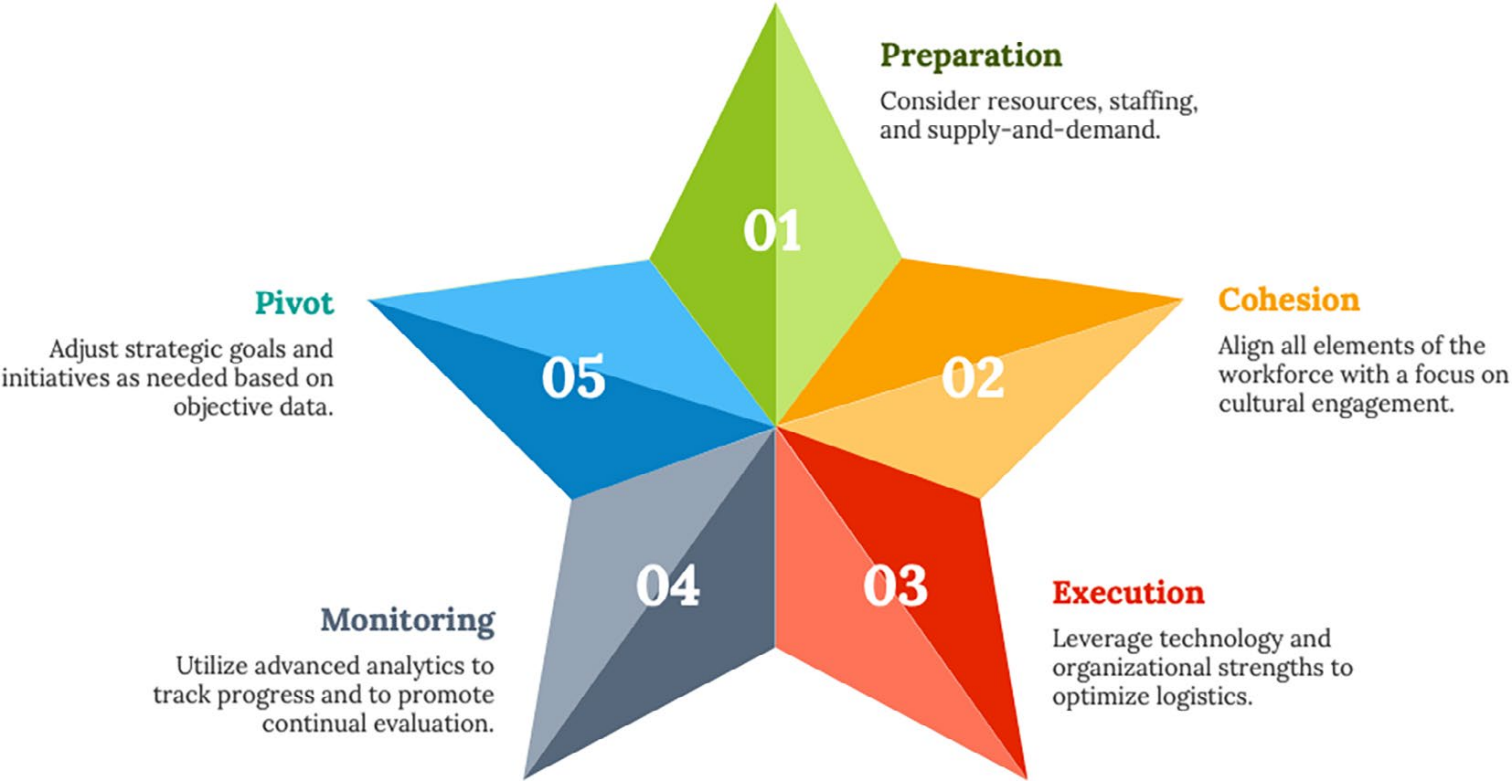
Illustrative framework of the themes outlined.

Due to the potentially disruptive nature of access improvement and the possibility of creating more strain on a healthcare system already overtaxed in many ways, the ability to plan and execute with deliberate focus (“be quick”) while exercising patience (“don't hurry”), in accordance with the principles espoused by Coach Wooden, is paramount. Given the multi‐faceted nature of access improvement and the complexities associated with its implementation, consistent leadership steadfastly committed to strategy, culture, engagement, change management, and attention to detail is imperative to making progress. Although the advantages of moving quickly and effortlessly are obvious, the fine line between doing so thoughtfully and hastily needs to always be in focus. To assuage this divide, the 5 empirical lessons reviewed above (Figure 1), focusing on the principles of strategy, alignment, execution, adaptation, and reflection, respectively, provide a tactical framework for placing access improvement in practical context—so that a precious balance between speed and deliberateness can be achieved. Ultimately, by thoughtfully addressing resistance to change while fostering a culture of continuous improvement and value transformation, access improvement has the potential to make meaningful and sustained gains benefiting all members of society—so that everybody on the “team” benefits.

## Author Contributions

A.C. is the sole author and is responsible for all facets of this work.

## Ethics Statement

This study was approved by the local Institutional Review Board (#2589) in accordance with the Declaration of Helsinki.

## Consent

A waiver was obtained for publication.

## Conflicts of Interest

The author declares no conflicts of interest.

## Data Availability

Data sharing not applicable to this article as no datasets were generated or analysed during the current study.
